# Dimensional changes of endodontic sealers—An in vitro model simulating a clinical extrusion scenario during 18 months

**DOI:** 10.1002/cre2.704

**Published:** 2023-01-11

**Authors:** Ankur Razdan, Ana R. Benetti, Azam Bakhshandeh, Tron A. Darvann, Lars Bjørndal

**Affiliations:** ^1^ Department of Odontology, Section of Dental Materials, Section of Clinical Oral Microbiology, Faculty of Health and Medical Sciences University of Copenhagen Copenhagen N Denmark; ^2^ Department of Odontology, Section of Cariology and Endodontics, Section of Clinical Oral Microbiology, Faculty of Health and Medical Sciences University of Copenhagen Copenhagen N Denmark; ^3^ Department of Dentistry and Oral Health, Section for Oral Radiology Aarhus University Aarhus C Denmark; ^4^ 3D Craniofacial Image Research Laboratory, School of Dentistry University of Copenhagen Copenhagen N Denmark; ^5^ 3D Craniofacial Image Research Laboratory, Centre of Head and Orthopaedics Copenhagen University Hospital Rigshospitalet Copenhagen N Denmark; ^6^ 3D Craniofacial Image Research Laboratory, Department of Applied Mathematics and Computer Science Technical University of Denmark Copenhagen N Denmark; ^7^ Department of Oral and Maxillofacial Surgery Copenhagen University Hospital Rigshospitalet Copenhagen N Denmark

**Keywords:** dental cements, in vitro techniques, root canal sealer, solubility

## Abstract

**Objectives:**

To examine the dimensional changes of endodontic sealers during 18 months using three‐dimensional (3D) surface scanning and subtraction radiography in a novel in vitro sealer‐extrusion model.

**Material and Methods:**

Fifty endodontically instrumented acrylic teeth were randomly allocated to five groups (*n* = 10) filled with Apexit Plus, AH Plus, BioRoot RCS, TubliSeal EWT, and gutta‐percha (control). Freshly mixed sealers were intentionally extruded during obturation. All teeth were immersed in a physiologic solution for up to 18 months. Blinded 3D surface scans (resolution: ~10 μm) and digital radiographs of the teeth were obtained at baseline (immediately after obturation); and then after 1 week, and at 1, 3, and 18 months. For blinded assessment of sealer dimensional change, 3D models and radiographs were superimposed using specific software. Volumetric differences, root mean square (RMS), and area change from subtraction radiography measured at each period within each sealer group were thereafter calculated. Repeated measures analyses were done with Bonferroni adjustment for multiple comparisons; standard errors, *p* values, and 95% confidence intervals (CI) were reported.

**Results:**

Analyses of the volumetric data confirmed significant, progressive material loss for Apexit Plus when compared to the other investigated sealers or the control group (*p* ≤ 0.02). Immersion period significantly influenced the volumetric dimensional changes of Apexit Plus already after 1 month (*p* < 0.01). For TubliSeal EW, the effect of the immersion period on the dimensional changes was noted after immersion for 3 months (*p* ≤ 0.02), while for BioRoot RCS this was evident only at 18 months (*p* < 0.01). Same trends were noted for the RMS data, whereas progressive dimensional changes using subtraction radiography only revealed significant changes for Apexit Plus (*p* = 0.01).

**Conclusions:**

The largest dimensional changes were shown by Apexit Plus, followed by Tubliseal EWT and BioRoot RCS. AH Plus remained stable throughout 18 months.

## 1 INTRODUCTION

Currently, standard tests are used for in vitro assessment of the solubility of endodontic root canal sealers (American National Standards Institute and American Dental Association Council on Scientific Affairs, 2012; ISO 4049, 2009; ISO 6876, 2012). While these tests provide a benchmark for accepted quality in terms of material properties and biological compatibility, their results may be difficult to relate to clinical practice. Some of the methodological issues associated with the standard tests are the evaporation of the mixing liquid during the drying of the sample (Vivan et al., 2010), the use of water for immersion media instead of body fluids (Gandolfi et al., 2016), and the fluid uptake by set sealers (Grga et al., 2011). Additionally, current standard tests do not seem appropriate for calcium‐silicate‐based sealers, as their dimensional stability does not seem to comply with the tests advocated in international standards (De‐Deus et al., 2022). As these tests are often set up differently than the clinical procedures, the solubility values derived from them cannot easily be extrapolated to in vivo situations (De‐Deus et al., 2022; Razdan et al., 2019) such as sealer extrusion. Various authors have already highlighted the need for alternative methods for assessing sealer dissolution (Gandolfi et al., 2016; Grga et al., 2011; Ha et al., 2017; Silva et al., 2016; Vivan et al., 2010) and for detailed reporting on in vitro studies (Nagendrababu et al., 2019).

Current knowledge regarding the dimensional stability and behavior of endodontic sealers is based primarily on in vitro solubility studies. Although epoxy‐based sealers are known to have low solubility in water and to be relatively stable dimensionally (Duarte et al., 2010; Kazemi et al., 1993; Ørstavik, 1983; Ruiz‐Linares et al., 2013), they also have some net fluid uptake (Donnelly et al., 2007; Grga et al., 2011). Zinc oxide sealers show slight but steady dimensional loss (McMichen et al., 2003), while calcium hydroxide sealers are prone to progressive dissolution (Grga et al., 2011; McMichen et al., 2003). The more recent hydraulic calcium‐silicate‐based sealers have been found to uptake fluid (Prüllage et al., 2016), release calcium, and nucleate calcium phosphate precipitates on the surface that is in contact with the surrounding media (Siboni et al., 2017; Xuereb et al., 2015). However, the findings described above on the likely solubility behavior of sealers were all derived from limited, short‐ and medium‐term data. Long‐term studies are required to improve the understanding of sealer dynamics, which are especially relevant in situations involving sealer extrusion. Such studies may contribute to understanding the role of sealers' dimensional characteristics in the outcome of endodontic treatment. Based on a moderate level of evidence, a recent review concluded that clinical sealer extrusion is more likely than no extrusion to contribute to a nonhealing outcome (Aminoshariae & Kulild, 2020).

Thus, the aim of the present study was to propose a novel in vitro model that simulates endodontic sealer extrusion including viable clinical parameters. Three‐dimensional (3D) surface‐scanning and digital subtraction radiography were employed to analyze the dimensional changes of distinct endodontic sealers over a period of 18 months. It was hypothesized that the in vitro method would be able to identify differences between dimensional changes in various endodontic sealers extruded over an extended period.

## 2 MATERIALS AND METHODS

### 2.1 Study design

**Figure 1:**
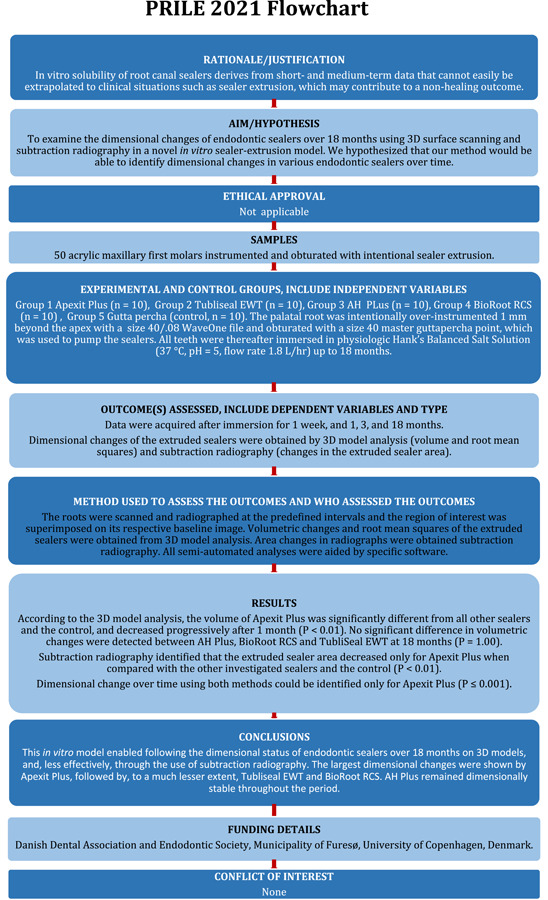
Preferred Reporting Items for Laboratory studies in Endodontology (PRILE) 2021 flowchart

**Figure 2:**
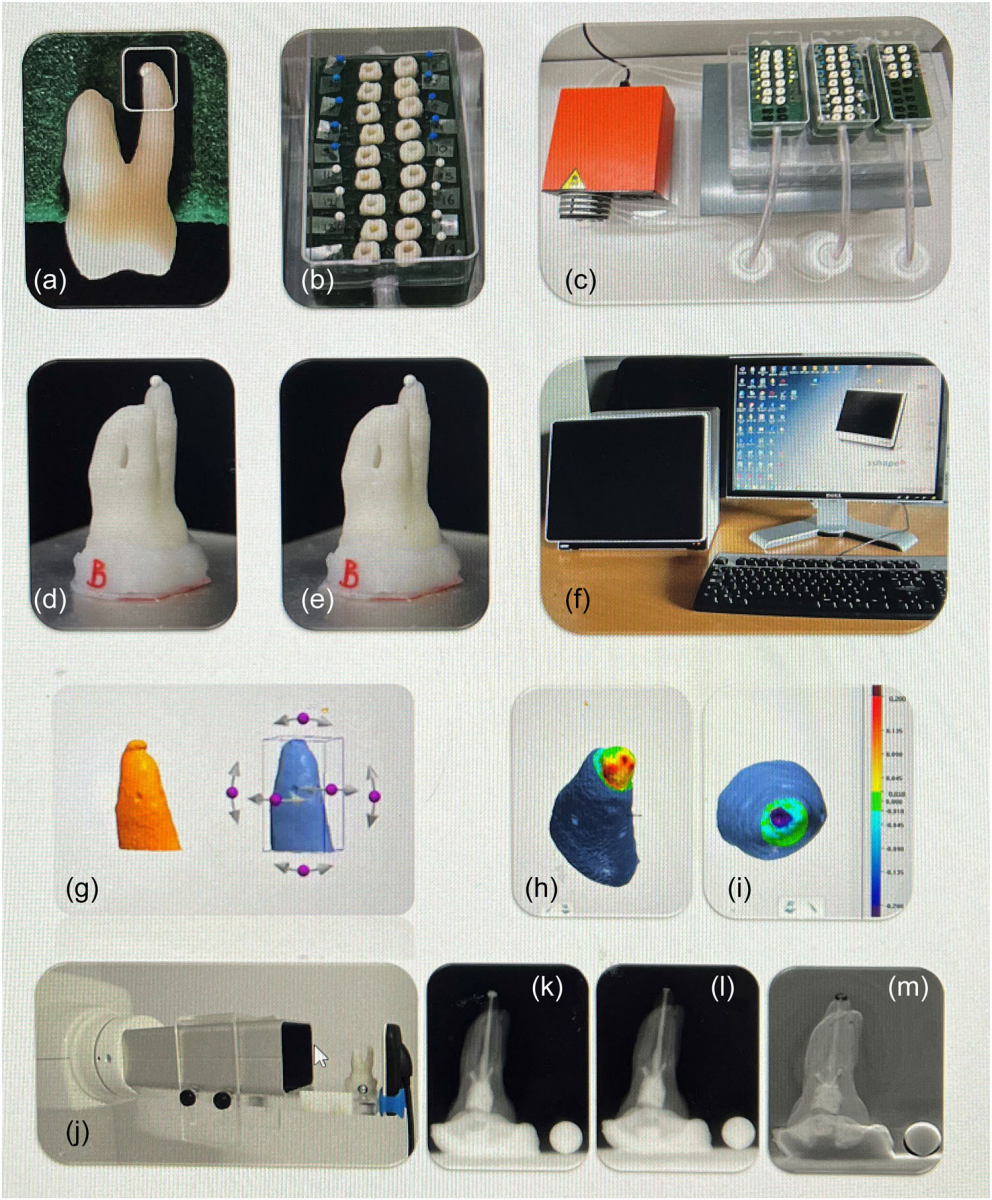
Overview of the study methods. Root with extruded sealer in polyurethane frame (a), immersed in Hank's Balanced Salt Solution (b), and attached to pump (c). Root with an extruded sealer at baseline (d) and follow‐up (e) (same photo shown to reflect that it was the same specimen used) mounted on holder for three‐dimensional scanning (f). Models were superimposed using software (g) to assess dimensional increase (h) or decrease (I). Root mounted on holder for digital radiography (j). Baseline (k) and follow‐up radiograph (l) were subtracted, and the area change was measured on the resulting image (m).

The manuscript of this laboratory study was written according to Preferred Reporting Items for Laboratory studies in Endodontology (PRILE) 2021 guidelines (Nagendrababu et al., 2021); an overview can be seen in Figure 1. An in vitro model was created to simulate a clinical extrusion scenario for testing dimensional changes in endodontic sealers (Figure 2). In relation to the status of extruded sealer dimension at baseline, the testing period was set to 1 week and 1, 3, and 18 months.

### 2.2 Samples

Power calculation confirmed that, when 3D surface scanning was used, nine samples in each group were needed to detect a 33% difference in mean dimensional change with a standard deviation estimate of 20%, at a two‐site *α* level of 5% (Type I error) and 90% power (Type II error of 10%). To allow for the possibility of a 10% loss of specimens, a final number of 10 samples was decided for each group, thereby following an earlier recommendation (Razdan et al., 2019).

For standardized specimen preparation, 50 acrylic endodontic‐training models of maxillary first molars (Endo Training Model; Dentalike, Dentsply Maillefer, Switzerland) with open‐access coronal cavities were used. These were then divided into five groups. The palatal roots in the four experimental groups were marked externally with three orifices and obturated using one of the following sealers: Apexit Plus (Ivoclar Vivadent, Schaan, Liechtenstein); TubliSeal EWT (Kavo Kerr Dental; Brea, CA, USA); AH Plus (Dentsply DeTrey, Konstanz, Germany); and BioRoot RCS (Septodont, Saint‐Maur‐des‐fossés Cedex, France). The sealers (Table 1) were mixed and used according to the manufacturer's instructions. In the control group, a size 40 WaveOne gutta‐percha point (Dentsply Sirona, Dentsply Maillefer, Switzerland) was placed without sealer. Table 1 shows the product details of the endodontic sealers tested.

**Table 1:** Endodontic sealers used in this study, lot numbers and composition stated by the manufacturers

Material	Manufacturer	Lot no.	Ingredients (weight, %)
AH Plus Paste A 4 ml Paste B 4 ml	Dentsply DeTrey GmbH, Germany	LOT 1611000530 LOT 1611000529	Paste A: Bisphenol‐A epoxy resin, bisphenol‐F epoxy resin, calcium tungstate, zirconium dioxide, silica, iron oxide pigments. Paste B: Dibenzyldiamine, aminoadamantane, tricyclodecane‐diamine, calcium tungstate, zirconium oxide.
Apexit Plus 6 g	Ivoclar Vivadent AG FL‐9494, Schaan, Liechtenstein	LOT V36978	Base: Calcium hydroxide/calcium oxide 36.9%; hydrated colophonium 54.0%; fillers and other auxiliary materials (highly dispersed silicon dioxide, phosphoric acid alkyl ester) 9.1%. Activator: Disalicylate 47.6%; bismuth hydroxide/bismuth carbonate 36.4%; fillers and other auxiliary materials (highly dispersed silicon dioxide, phosphoric acid alkyl ester) 16.0%.
BioRoot RCS Powder 15 g Liquid 0.2 ml	Septodont CO 80027, USA	LOT B17315	Powder based on tricalcium silicate, zirconium oxide and povidone. Liquid based on calcium chloride and polycarboxylate solution.
Tubli‐Seal EWT Base 15 g Accelerator 5 g	Kerr Corporation, 48174, MI, USA	LOT 5‐1257	Base: Zinc oxide (60%–100%); white mineral oil (10%–30%). Accelerator: Eugenol (10%–39%); white mineral oil (5%–10%).

#### 2.2.1 Tooth preparation

As the buccal root tips of each tooth were not used in this investigation, they were sealed using a light‐cured resin (UltraSeal XT Plus, Ultradent Products Inc., Cologne, Germany). The palatal root was intentionally over instrumented. The proper working length (WL) was 21.5 mm from the palatal reference cusp (to remain 1.0 mm away from the anatomical apex visualized on the radiograph). However, a final WL of 22.5 mm was used to enable intentional “standardized” overinstrumentation. A size 40/0.08 WaveOne file (Dentsply Sirona, Dentsply Maillefer) was used for biomechanical instrumentation of the canal.

#### 2.2.2 Randomization and concealed allocation

After endodontic preparation, a concealed‐allocation sequence was used to randomly allocate each palatal root to one of the five groups (*n* = 10) and was carried out by the main investigator (A. R.).

### 2.3 Intentional sealer extrusion procedure

The application of the extruded sealer was controlled in each palatal root by manually pumping the sealer five times in an anticlockwise direction using a size 40 WaveOne master gutta‐percha point (Dentsply Sirona, Dentsply Maillefer). This allowed a similar extrusion pattern in all samples. Obturation with the master point adjusted by cutting 1 mm apically was followed by lateral condensation using two accessory cones (size C, Dentsply Sirona, Dentsply Maillefer). The resulting volume and exposed surface area of the sealer were compatible with those in a clinical apical extrusion scenario (Figure 2a). As the investigator needed to identify the sealer types during their manipulation and use, it was not possible to blind each root‐filling procedure. In the control group without sealer, each palatal root was instrumented as previously described and an intact gutta‐percha cone was intentionally extruded. A flowable composite (Venus Bulk Fill, Hanau, Germany) was used to fill the access cavity over the canal orifices.

#### 2.3.1 Tooth immersion

Throughout the experiment, the acrylic teeth were placed in polyurethane frames that were immersed in physiologic Hank's Balanced Salt Solution (HBSS) (The Substrate and Sterile Laboratory, University of Copenhagen, Copenhagen, Denmark) at pH 7 and 37°C. Using an assembly pump (Type 110; Ole Dich Instrumentmakers Aps, Hvidovre, Denmark), a prespecified quantity of HBSS (500 ml) was put into circulation through the polyurethane frames (Figure 2b) at a fixed flow rate of 1.8 ml/h (Figure 2c). To avoid material saturation or the growth of microorganisms, the solution was changed weekly during the entire test period.

The roots were immediately immersed after obturation when the extruded sealer was not set. The immersed roots were removed from the set‐up for subsequent 3D scanning and radiographs only at the predetermined intervals (Figure 2d,e). The surface of the extruded sealer was gently rinsed with HBSS. Each time, the roots were transported fully immersed in HBSS.

### 2.4 Assessment of dimensional changes of sealer

Blinded 3D models and digital radiographs of the palatal roots were obtained at baseline, 1 week, and 1, 3, and 18 months. At baseline, the acquisition of the 3D model of each palatal root and the digital radiographic images occurred within 2 h of obturation.

#### 2.4.1 3D surface models

The 3D models were obtained by scanning the palatal roots (Figure 2f,g) with a table scanner (Convince ScanWizard, Model Q800 SN: K1049004B; 3Shape, Copenhagen, Denmark) at a resolution of ~10 μm and using scanning software (Convince 2015, version 3.0.2.2; 3Shape). Fixed predefined settings were used during the scanning procedure. To scan the palatal root, 10 scans per camera swing (fixed at 0°, 40°, and 80°) were acquired. A calibration block was used for the daily calibration of the scanner.

#### 2.4.2 Digital radiographs

The digital radiographs were obtained using an intraoral X‐ray (Figure 2j–l) unit at 8 mA and 6 KV (Planmeca ProSensor® HD; Planmeca ProX, Helsinki, Finland), and specific software (Romexis, Planmeca OY, Helsinki, Finland). The exposure time was 0.5 s. These radiographs were taken for WL determination, master‐cone placement, postoperative baseline (postobturation of freshly extruded sealer within the first 2 h), and follow‐up assessments. The alignment of the palatal root was standardized using an accessory aiming to take period‐identical radiographs (Figure 2j). Measurements were calibrated using a stainless‐steel calibration ball (5 mm in diameter) for the digital radiographs (Figure 2j–m).

### 2.5 Data analysis

Specific coding was used to blind the investigators to the sealer groups. The main investigator (A. R.) obtained all 3D models and two‐dimensional (2D) digital radiographs. Image analyses were performed by the main investigator in collaboration with the other investigators.

#### 2.5.1 Analysis of 3D models

Dimensional changes in the extruded sealer over time were acquired using two specific software. For both software, the subsequent 3D models (in Standard Tessellation Language [STL] file‐format) obtained at various readings were superimposed on the baseline models. The region of interest (ROI) for model alignment was defined by the palatal periapical region and the extruded sealer.

**Figure 3:**
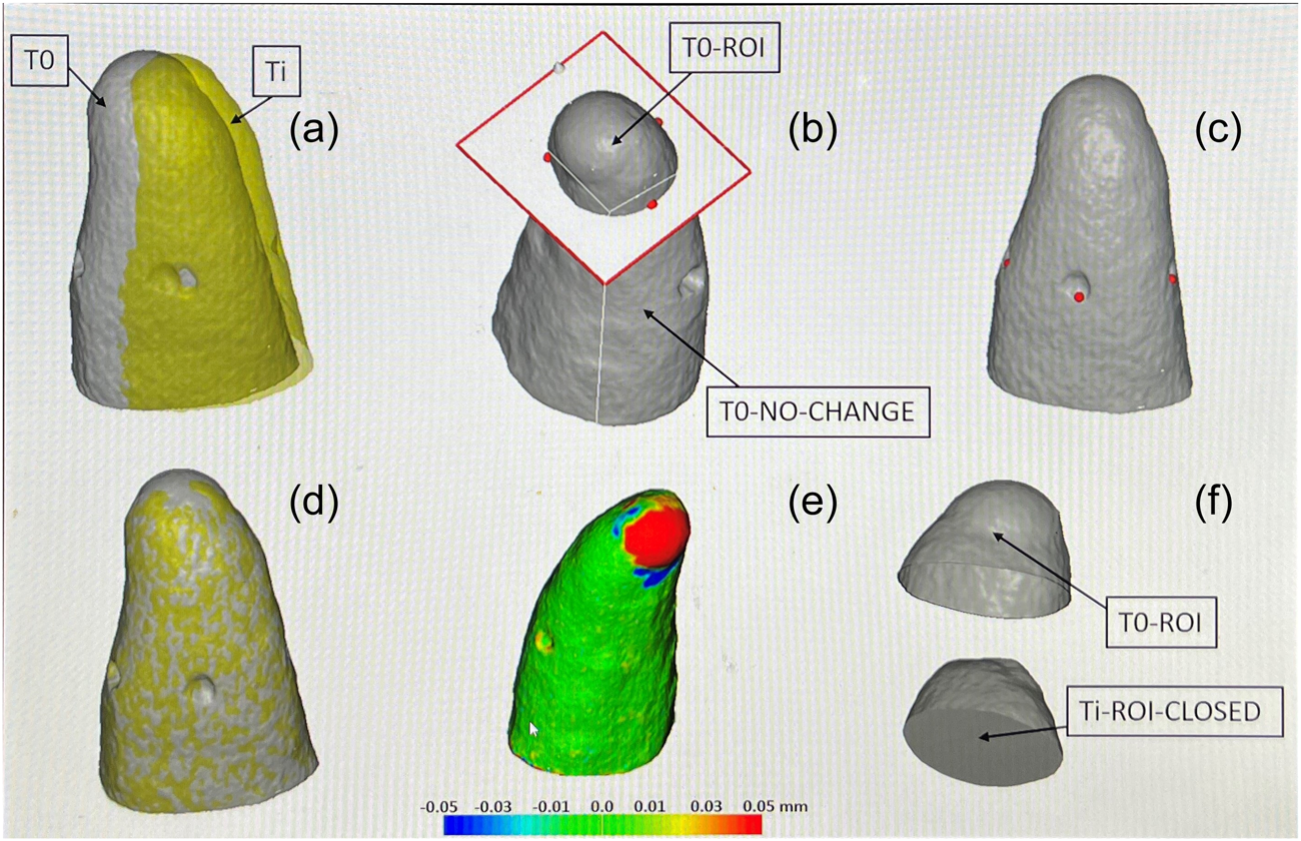
Illustration of measurement of temporal volume change in a palatal root. (a) Two scans of a palatal root at two different time points: Baseline scan (T0) (gray surface) and a scan at a later time point (Ti) (yellow surface). (b) On T0, a cutting plane is defined that separates the root surface into two parts: T0‐region of interest (ROI) which is an ROI where the sealer is located and hence will potentially experience a volume change, and T0‐NO‐CHANGE, which is the rest of the surface where by definition no change will occur. (c) Illustration of the location of three landmarks that were manually placed on both surfaces (T0 and Ti), used as input to an initial rigid landmark‐based registration of Ti relative to T0. (d) Result of an iterated closest point (ICP) final registration applied after the landmark‐based initialization. (e) Illustration of distances (in mm according to the color bar) between the two surfaces after the final registration, indicating the amount and location of change. (f) Due to the registration, the cutting plane in (b) will cut the T0 and Ti surfaces in the exact same location on the two surfaces. Volumes V0 and Vi of the corresponding ROIs are calculated after closing the ROI surfaces (the bottom figure shows Ti‐ROI after closure). The volume change is the difference V0–Vi.

Volumetric data were extracted using software (Landmarker version 2.0.9a 2019, University of Copenhagen). This software was tailored to identify the temporal volumetric change in a small ROI using an iterated closest point algorithm (ICP) based on a set of landmarks (Figure 3). The periapical ROI was separated from the rest of the digital model at a level with the landmarks. The hollow ROI model mesh (i.e., STL file) was then closed using another software (Meshmixer version 3.5.474, 2017; Autodesk Inc., San Rafael, CA, USA). The quality of the alignment was inspected, and the volume of the ROI model was then calculated using the Landmarker software (Appendix A). Volumetric differences between ROI models obtained at each observation period were thereafter calculated.

Root mean square (RMS) values were obtained using proprietary software (Convince version 2015; 3Shape). The RMS value at the ROI was obtained after models were superimposed (Figure 2g) using a best‐fit algorithm. Because an RMS represents the mean of all squared distances between multiple points on the overlapping models after the square root has been taken, the RMS value accounts for the absolute offset between 3D models obtained at each follow‐up when superimposed on its corresponding baseline model. The profilometric RMS expresses the magnitude of change without indicating any dimensional increase (which would be given by positive values), or any decrease (which would be given by negative values). The mean dimensional change in sealer (mm) was visualized by the color map (Figure 2h,i) in the software (Convince 2015; 3Shape). According to the color map for the predefined threshold, green signified no change, yellow and red signified a dimensional increase (Figure 2h), while blue and purple signified a dimensional decrease (Figure 2i).

#### 2.5.2 Subtraction radiography analysis of 2D digital radiographic images

All radiographic images were saved in tiff format at a bit size of 8 grayscale. After ensuring that each image had a uniform width of 620 pixels in the bitmap format, ImageTool software (version 1.23; University of Texas Health Science Centre, San Antonio, TX, USA) was used to subtract each radiograph (Figure 2m) obtained at the predefined intervals (Figure 2l) from the baseline radiograph (Figure 2k). The ROI for comparison was chosen in a similar way as that for the 3D analysis. Any differences in sealer area (mm^2^) for each individual specimen were calculated at different time intervals in relation to its corresponding baseline radiograph.

### 2.6 Stereomicroscopic images

To show the macroscopic surface of the extruded sealers, photographs (InfinityX camera; Dentalpix, Canada) were taken at 3 and 18 months under a stereomicroscope (magnification ×2.5) (SteREO, Discovery V8; Carl Zeiss Microscopy, Jena, Germany).

### 2.7 Outcome measures

The primary outcome measures were the volumetric dimensional changes of the extruded sealers over time. For the 3D models, the profilometric RMS values between the baseline and the subsequent follow‐up models were also included. For subtraction radiography, differences in sealer area between the baseline and subsequent radiographs were calculated.

#### 2.7.1 Measurement uncertainty

Measurement uncertainties (*u*) associated with each method were calculated based on the mean and standard deviations from 10 overlaps on the same image using the formula.

u=S/√n,

where *S* is the sample standard deviation and *n* is the number of measurements in the set. By multiplying the measurement uncertainty by a coverage factor *k* = 2, the standard error (SE) of the mean was provided within a level of confidence of 95%.

### 2.8 Statistical methods

The data collected over the various assessment periods were analyzed as repeated measures using PROC MIXED in SAS 9.4 (SAS Institute, Cary, NC, USA) with either volumetric dimensional changes, RMS, or area change using subtraction radiography as the response variables; sealer group and time as fixed effects; and a compound symmetry covariance structure for the response variable. Multiple comparisons were made using the Bonferroni adjustment. Similar models were created for each sealer group separately to compare the volumetric dimensional change, RMS, and area change from subtraction radiography measured at each period within each group. Least squares (LS) means between the groups and the assessment periods, with corresponding SE, *p* values, and 95% confidence intervals (CI) were obtained from these models. The overall interaction of each sealer with each assessment period was analyzed for volumetric dimensional changes, RMS, and area change from subtraction radiography.

## 3 RESULTS

**Figure 4:**
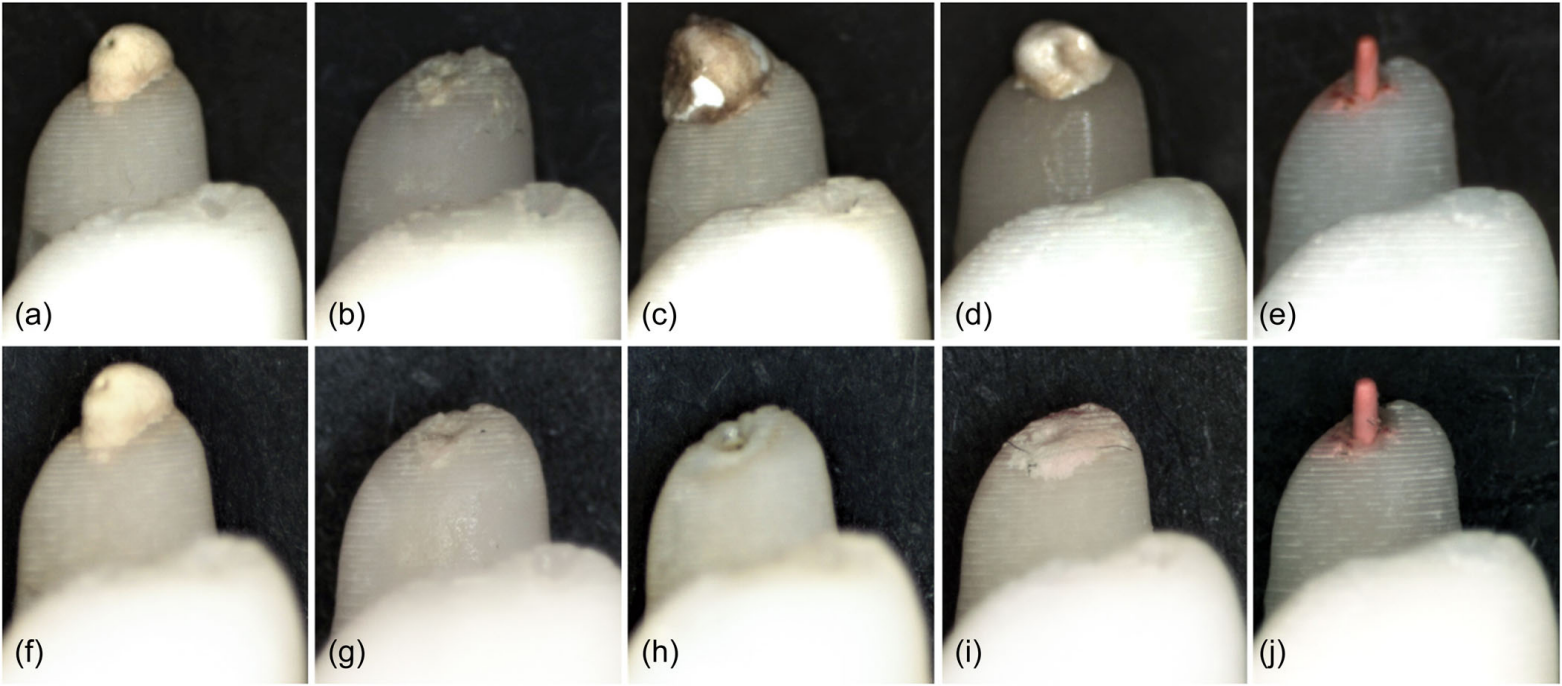
Illustration of macroscopic changes (magnification ×2.5). Pairwise photographs of sealers after 3 months (top row) and 18 months (bottom row) immersed in Hank's Balanced Salt Solution: AH Plus with stable appearance (a, f). Apexit Plus already reduced at 3 months (b, g). BioRoot RCS showing detachment at 18 months (c, h). TubliSeal EWT reduced at 18 months (d, i). Gutta‐percha remains stable (e, j).

Due either to inappropriate digital model alignment or loss of specimens, full data could not be acquired for five roots (1 AH Plus, 1 Apexit Plus, 1 BioRoot RCS, 2 Tubliseal EWT). For three additional BioRoot RCS specimens, detachment of the sealer from the root apex occurred before the 3‐month reading. Figure 4h shows a detachment case at 18 months.

Due either to inappropriate radiograph alignment or loss of specimens, no subtraction radiography data could be obtained from nine roots at 18 months (2 AH Plus, 1 Apexit Plus, 4 BioRoot RCS, 2 Tubliseal EWT).

Significant differences were observed between sealer groups (*p* < 0.05), assessment periods (*p* < 0.05) and their interaction (*p* < 0.05) for data resulting from the digital model analyses (i.e., volumetric change and RMS) and subtraction radiography. LS mean differences for the dimensional changes among sealers are displayed in Table 2, while the effect of immersion period on the dimensional changes of each sealer is visualized in Table 3.

**Table 2:** Differences of least squares means values of endodontic sealers after immersion for 18 months

	Volumetric difference (mm^3^)				Root mean square (mm)				Subtraction radiography (mm^2^)			
Effect	Estimate	SE	*p*	95% CI	Estimate	SE	*p*	95% CI	Estimate	SE	*p*	95% CI
AH versus AX	0.76	0.15	<0.01*	0.32, 1.20	−0.08	0.02	<0.01*	−0.13, −0.03	1.27	0.18	<0.01*	0.73, 1.81
AH versus BR	0.18	0.15	1.00	−0.26, 0.62	−0.05	0.02	0.08	−0.09, 0.00	0.14	0.19	1.00	−0.42, 0.69
AH versus TS	0.26	0.15	1.00	−0.20, 0.71	−0.02	0.02	1.00	−0.06, 0.03	0.44	0.18	0.20	−0.10, 0.98
AH versus GP	0.03	0.15	1.00	−0.41, 0.47	0.03	0.02	0.74	−0.02, 0.07	0.19	0.18	1.00	−0.35, 0.72
AX versus BR	−0.58	0.14	<0.01*	−1.01, −0.15	0.03	0.02	0.38	−0.01, 0.08	−1.14	0.18	<0.01*	−1.68, −0.60
AX versus TS	−0.50	0.15	0.02*	−0.94, −0.06	0.06	0.02	<0.01*	0.02, 0.11	−0.84	0.18	<0.01*	−1.36, −0.31
AX versus GP	−0.73	0.14	<0.01*	−1.15, −0.30	0.11	0.02	<0.01*	0.06, 0.15	−1.09	0.18	<0.01*	−1.61, −0.57
BR versus TS	0.08	0.15	1.00	−0.36, 0.52	0.03	0.02	.67	−0.02, 0.08	0.30	0.18	1.00	−0.24, 0.84
BR versus GP	−0.14	0.14	1.00	−0.57, 0.28	0.07	0.02	<0.01*	0.03, 0.12	0.05	0.18	1.00	−0.49, 0.59
TS versus GP	−0.22	0.15	1.00	−0.66, 0.22	0.04	0.02	.06	0.00, 0.09	−0.25	0.18	1.00	−0.78, 0.27

*Note*: Estimate, SE, *p* value, and 95% CI after Bonferroni adjustment are presented for volumetric difference, RMS, and subtraction radiography.

Abbreviations: AH, AH Plus; AX, Apexit Plus; BR, BioRoot ACS; CI, confidence interval; GP, gutta‐percha; RMS, root mean square; SE, standard error; TS, TubliSeal EW.

* *p* < 0.05.

**Table 3:** Differences of least squares means for the effect of immersion time on each sealer in relation to baseline

	Volumetric difference (mm^3^)				Root mean square (mm)				Subtraction radiography (mm^2^)			
Effect	Estimate	SE	*p*	95% CI	Estimate	SE	*p*	95% CI	Estimate	SE	*p*	95% CI
AH 1 week	0.02	0.14	0.90	−0.25, 0.29	0.05	0.02	<0.01*	0.01, 0.08	0.04	0.16	0.79	−0.27, 0.36
AH 1 month	0.00	0.14	0.98	−0.27, 0.27	0.04	0.02	<0.01*	0.01, 0.08	0.23	0.16	0.14	−0.08, 0.55
AH 3 months	−0.03	0.14	0.83	−0.30, 0.24	0.05	0.02	<0.01*	0.01, 0.08	0.20	0.16	0.21	−0.12, 0.51
AH 18 months	0.03	0.14	0.83	−0.25, 0.31	0.05	0.02	<0.01*	0.02, 0.09	0.34	0.16	0.04*	0.01, 0.66
AX 1 week	−0.23	0.13	0.09	−0.48, 0.03	0.06	0.02	<0.01*	0.02, 0.09	−0.44	0.15	<0.01*	−0.74,−0.14
AX 1 month	−0.60	0.13	<0.01*	−0.86, −0.35	0.12	0.02	<0.01*	0.09, 0.15	−1.05	0.15	<.01*	−1.35, −0.75
AX 3 months	−1.07	0.13	<0.01*	−1.33, −0.81	0.17	0.02	<0.01*	0.14, 0.20	−1.43	0.15	<0.01*	−1.73, −1.13
AX 18 months	−1.11	0.13	<0.01*	−1.38, −0.84	0.17	0.02	<0.01*	0.13, 0.20	−1.37	0.15	<0.01*	−1.67, −1.06
BR 1 week	0.24	0.13	0.08	−0.03. 0.50	0.10	0.02	<0.01*	0.07, 0.13	0.20	0.15	0.19	−0.102, 0.50
BR 1 month	0.00	0.13	0.99	−0.25, 0.26	0.08	0.02	<0.01*	0.04, 0.12	0.13	0.17	0.44	−0.21, 0.47
BR 3 months	−0.26	0.13	0.05	−0.51, 0.00	0.08	0.02	<0.01*	0.04, 0.12	0.02	0.17	0.92	−0.32, 0.35
BR 18 months	−0.67	0.13	<0.01*	−0.93, −0.40	0.11	0.02	<0.01*	0.07, 0.15	−0.08	0.18	0.68	−0.43, 0.28
TS 1 week	−0.11*	0.14	0.41	−0.38, 0.16	0.07	0.02	<0.01*	0.04, 0.10	−0.15	0.15	0.32	−0.45, 0.15
TS 1 month	−0.12	0.14	0.39	−0.39, 0.15	0.06	0.02	<0.01*	0.03, 0.09	−0.31	0.15	0.04*	−0.61, −0.01
TS 3 months	−0.33	0.14	0.02*	−0.60, −0.06	0.05	0.02	<0.01*	0.02, 0.08	−0.36	0.16	0.02*	−0.67, −0.05
TS 18 months	−0.44	0.14	<0.01*	−0.72; −0,16	0.08	0.02	<0.01*	0.04, 0.11	−0.12	0.16	0.47	−0.44, 0.20
GP 1 week	−0.02	0.13	0.87	−0.28, 0.24	0.02	0.02	0.16	0.00, 0.05	0.01	0.15	0.93	−0.29, 0.31
GP 1 month	0.00	0.13	0.99	−0.26, 0.26	0.02	0.02	0.31	−0.02, 0.05	0.02	0.15	0.91	−0.28, 0.32
GP 3 months	−0.07	0.13	0.60	−0.33, 0.19	0.02	0.02	0.18	−0.01, 0.05	0.02	0.15	0.91	−0.28, 0.32
GP 18 months	−0.02	0.13	0.91	−0.28, 0.25	0.02	0.02	0.25	−0.01, 0.05	0.02	0.15	0.91	−0.28, 0.32

*Note*: Estimate, SE, *p* value, and 95% CI are presented for volumetric difference, RMS, and subtraction radiography.

Abbreviations: AH, AH Plus; AX, Apexit Plus; BR, BioRoot ACS; CI, confidence interval; GP, gutta‐percha; RMS, root mean square; SE, standard error; TS, TubliSeal EW.

* *p* < 0.05.

### 3.1 Assessment of 3D models

**Figure 5:**
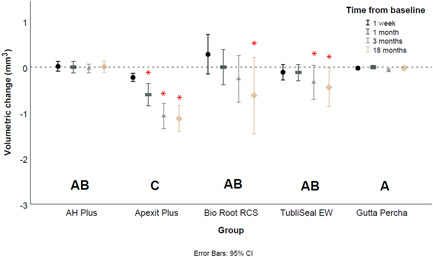
Mean volumetric change (mm^3^) and standard deviation of endodontic sealers at the region of interest over time. Error bars represent the 95% confidence intervals and asterisks indicate statistical significance in relation to the baseline.

**Figure 6:**
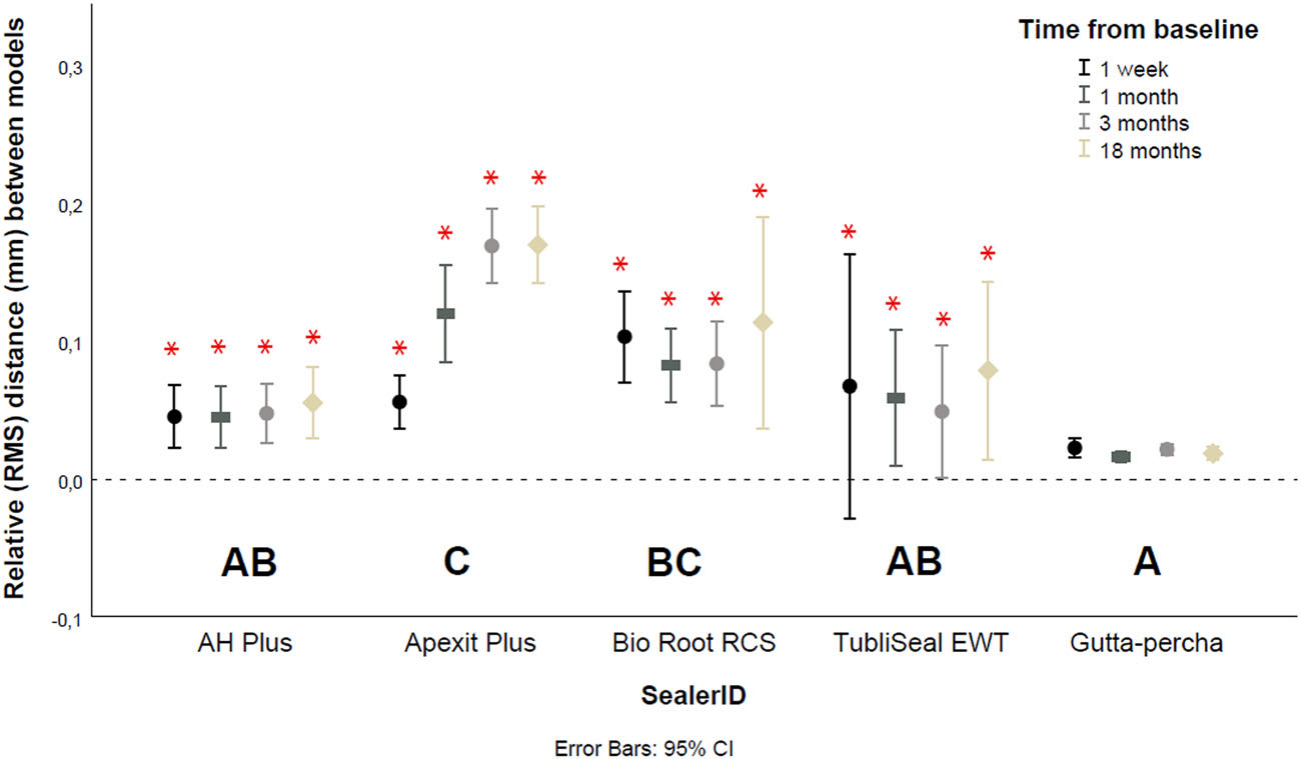
Mean root mean squares (mm) of sealers at region of interest over time. Error bars represent the 95% confidence intervals and asterisks indicate statistical significance in relation to the baseline.

At 18 months, the magnitude of dimensional changes between sealers differed significantly (*p* < 0.01, Figures 5 and 6). A slight increase in dimensions was observed for AH Plus after 1 week of immersion; thereafter, the material dimensions remained unchanged throughout the 18 months. On the contrary, a decrease in dimensions was noted for TubliSeal EW and Apexit Plus; the latter underwent a significant progressive dimensional decrease over time. Conversely, BioRoot RCS showed an immediate increase in dimensions after 1 week of immersion, followed by subsequent volumetric reduction. Within the measurement uncertainty of the current experiments, gutta‐percha remained stable.

The stereomicroscopic photographs show early trends for AH Plus (Figure 4a,f), Apexit Plus (Figure 4b,g), Tubliseal EWT (Figure 4d,i), and gutta‐percha (Figure 4e,j), but not for BioRoot RCS (Figure 4c,h), where sealer detachment in some specimens occurred later.

### 3.2 Volumetric assessments

Analyses of the volumetric data (Table 2, Figure 5) confirmed significant, progressive material loss for Apexit Plus when compared to the other investigated sealers or the control group (*p* ≤ 0.02). No significant volumetric differences were found between the remaining sealers and gutta‐percha (*p* = 1.00).

The immersion period significantly influenced the volumetric dimensional changes (Table 3) of Apexit Plus already after 1 month (*p* < 0.01). For TubliSeal EW, the effect of the immersion period on the dimensional changes was noted after immersion for 3 months (*p* ≤ 0.02), while for BioRoot RCS significant influence of the immersion period was evident only at 18 months (*p* < 0.01).

### 3.3 Relative distance between models

The same general trend was observed for the RMS analyses (Table 2, Figure 6). Relative to the control, RMS values at 18 months for Apexit Plus (*p* < 0.01) and BioRoot RCS (*p* < 0.01) differed significantly from those of gutta‐percha. No significant differences in RMS values between AH Plus (*p* = 0.74), Tubliseal EWT (*p* = 0.06) and the control were found.

No significant differences in RMS were observed between BioRoot RCS, AH Plus, and TubliSeal EWT (*p* ≥ 0.08). Apexit Plus was not significantly different than BioRoot RCS (*p* = 0.38), though it had a significantly higher RMS value than AH Plus (*p* < 0.01) and TubliSeal EWT (*p* < 0.01).

The immersion period had a significant effect on the RMS of all sealers already from the first week (*p* ≤ 0.01). Only the control group (GP) was not affected by the immersion period, (*p* ≥ 0.16) (Table 3).

### 3.4 2D digital subtraction radiography

After 18 months, the area difference was significantly larger for Apexit Plus than for the other sealers (*p* < 0.01). Subtraction radiography identified no significant differences relative to the control for AH Plus (*p* = 1.00), BioRoot RCS (*p* = 1.00), and TubliSeal EWT (*p* = 1.00). Neither were there any significant differences between AH Plus, BioRoot RCS, and TubliSeal EWT (*p* ≥ 0.20).

**Figure 7:**
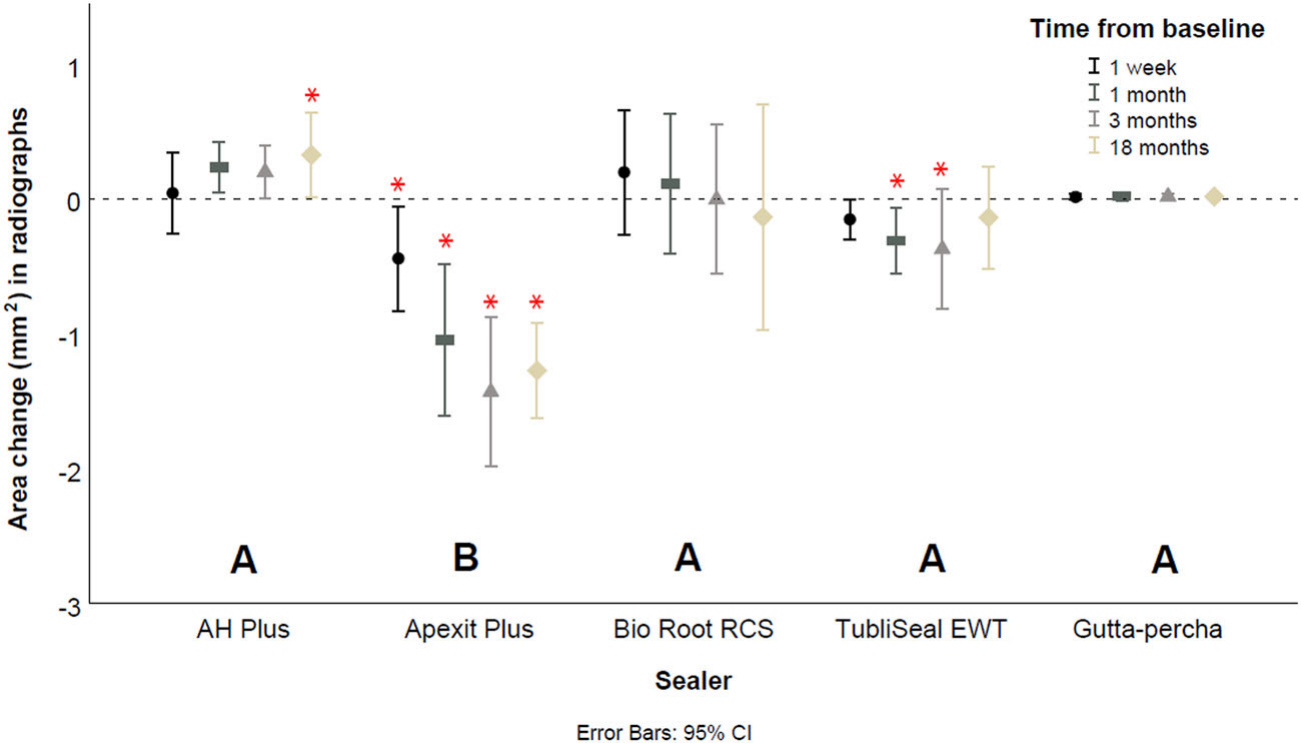
Mean area change (mm^2^) of endodontic sealers at the region of interest as shown by subtraction radiographs. Error bars represent the 95% confidence intervals and asterisks indicate statistical significance in relation to the baseline.

The immersion period had a significant effect on the sealer area obtained with subtraction radiography for AH Plus after 18 months (*p* = 0.04), as well as for TubliSeal EWT after 1 month (*p* = 0.04) and 3 months (*p* = 0.02). The only progressive dimensional change identified by subtraction radiography on all immersion periods was noted for Apexit Plus (Figure 7) (*p* = 0.01). The control group remained stable in the radiographs (Figure 7).

### 3.5 Report of measurement uncertainty

If the largest standard deviation from the sets of data is viewed as the worst‐case scenario for each method, measurement uncertainty for the 3D model assessments accounted for SE ± 0.036 mm^3^ from volumetric analyses, SE ± 0.027 mm for RMS measurements, and SE ± 0.167 mm^2^ for digital subtraction radiography, at a level of confidence of 95%.

## 4 DISCUSSION

This study presents the results of an 18‐month observation of the dimensional status of endodontics sealers, which, for the first time, used a novel in vitro model to simulate a sealer‐extrusion scenario. At a first glance, the model analyses showed that the dimensions of Apexit Plus and TubliSeal EWT had decreased after 1 week, while those of AH Plus and BioRoot RCS had increased (Figure 5). Thereafter, a progressive decrease in dimensions over time was observed for Apexit Plus, less evident dimensional changes were noted for Tubliseal EWT and BioRoot RCS, whilst AH Plus remained stable. These dimensional changes were significant—or not—according to the assessment method, that is, volumetric, profilometric (RMS), or radiographic (Table 3). As the proposed method could successfully identify the dimensional changes of endodontic sealers, the study hypothesis was accepted. It is especially worth to highlight the dimensional stability of the control group with very low variance. These long‐term data supplement those of previous short‐ and medium‐term in vitro studies (Kaplan et al., 1997; Kazemi et al., 1993; McMichen et al., 2003; Poggio et al., 2010; Rosa et al., 2010; Segato et al.,^2016^; Simões Filho et al., 1975; Urban et al., 2018).

Freshly mixed sealers were immediately immersed in the present study, as proposed by Bodanezi et al. (2008); since the sealers' setting can be delayed by immediate immersion in solution (Siboni et al., 2017), and the sealers' behavior may be affected by the immersion period (Prüllage et al., 2016). In addition, a physiologic solution (HBSS) was used, as recommended previously (Kebudi Benezra et al., 2017; Razdan et al., 2019), and the flow rate used in this study was similar to that of blood circulation. Overall, the 3D model analysis detected dimensional changes in extruded sealers better than subtraction radiography did, although a relatively large dispersion of the data was noted for the sealer groups. The following events are likely to have affected the higher‐than‐expected standard deviations. Changes in the shape of the extruded sealer observed during the reassessment periods were caused either by the low viscosity of the sealer or by abrupt material loss. One low‐viscosity sealer, in particular, Apexit Plus, dribbled over the lateral surface of the root, making it difficult to superimpose the subsequent models on the baseline model. A distinct problem observed for BioRoot RCS was that, at the later stages of the assessment, larger amounts of the extruded sealer were either washed out as previously assumed (De‐Deus et al., 2022) or were completely detached (Figure 4c,h). Although one could argue that this study has been impaired due to the loss of specimens resulting from the detachment of sealers, disregarding the disintegration of this sealer in an aqueous solution may in theory lead to underestimating its implications in vivo. Based on this experience, it is recommended that future studies are conducted and include more specimens per group.

As radiographs are the tools most commonly available to dentists in the clinical endodontic practice, it is very relevant to know whether they enable a dentist to follow‐up on changes in radiopacity and to detect dimensional changes in sealers over time (Ha et al., 2017). Even though this study aimed to obtain period‐identical radiographs, it was difficult in some cases to achieve perfect alignment between radiographs acquired at different time intervals. The comparison of radiographs is also influenced by factors such as acquisition angle and exposure time, which influence the overall image quality. Subtraction radiography identified substantial dimensional changes only for Apexit Plus but was not sensitive enough to detect gradual dimensional changes for the other sealers at the investigated intervals. Such findings suggest that routine radiographs are not ideal to assess sealer changes in a clinical setting.

When comparing the sealers, a discrete initial increase in dimension was observed for AH Plus, which can be explained by a net fluid uptake (Donnelly et al., 2007; Grga et al., 2011; Kebudi Benezra et al., 2017). AH Plus has previously been shown to have low solubility in water (Duarte et al., 2010; Kazemi et al., 1993; Ørstavik 1983; Ruiz‐Linares et al., 2013; Silva, Cardosa, et al., 2021) that has been attributed to strong cross‐links in the resin polymers (Arias‐Moliz et al., 2015; Borges et al., 2012). Within the confinements of the canal walls, low solubility is advantageous for a tight seal. However, it is speculated that the extruded epoxy‐based sealers may act as a stable surface for secondary biofilm proliferation. This may in turn sustain apical pathosis, as also suggested in a recent meta‐analysis on the impact of sealer extrusion on endodontic outcomes (Aminoshariae & Kulild, 2020). To confirm this claim, however, high‐quality studies are required. These should include appropriate control groups and conduct a precise pretreatment diagnosis on the periapical region of the teeth.

Unlike AH Plus, the calcium‐hydroxide‐based sealer, Apexit Plus, showed reduced dimensions that could be visualized macroscopically (Figure 4b,g). This dimensional decrease was progressive after 1 month, thereby confirming previous data (Grga et al., 2011; McMichen et al., 2003). Although some degree of solubility is desirable for calcium‐ion‐releasing sealers, high solubility may allow gaps to form between the sealer and the dentine walls, which, over time, are likely to allow intracanal bacterial growth (Ørstavik, 2005). Interestingly, the setting time for Apexit Plus seemed to be longer in HBSS, which may have favored sealer washout at the initial stages.

During the first week, the dimensions of the calcium‐silicate‐based sealer BioRoot RCS increased (Table 2). While this initial increase can be explained partly by the formation of surface precipitates resulting from the reaction of leached calcium ions with the immersion media (Siboni et al., 2017; Xuereb et al., 2015), the main explanation is fluid uptake (Kebudi Benezra et al., 2017; Prüllage et al., 2016) from hydrophilic nanosized particles within the sealer (Borges et al., 2012). As the literature does not provide enough evidence to substantiate the hypothesis that in vitro surface precipitation—often referred to as “bioactivity”—is also reflected in vivo, such precipitation is most likely misinterpreted as a sign of in vivo bioactivity (Tay, 2014). The present results on BioRoot RCS confirm that the dimensional increase in this sealer is of nonbiological origin. Additionally, in 3 out of 10 specimens in this study, the extruded calcium‐silicate‐based sealer detached from the roots. The detachments noted in BioRoot RCS may have resulted from its hydraulic nature (Formosa et al., 2013), which possibly led to premature disintegration when this sealer was in contact with fluids. A recent meta‐analysis confirmed higher solubility of several calcium silicate‐based sealers—including BioRoot RCS—when compared to the resin‐based AH Plus (Silva, Cardosa, et al., 2021). It remains to be investigated whether these findings represent a weakness that may compromise the provision of a long‐term bacteria‐tight seal.

A slight, initial decrease in the dimensions of the zinc oxide sealer Tubliseal EWT was noted, as shown previously (McMichen et al., 2003), but significant material loss over time could be confirmed from 1 month onward. Earlier observations of periapically extruded sites during clinical radiographic controls over time (Ricucci et al., 2016) corroborate such findings for this sealer. Thus, the clinical relevance of using radiographs as tools—either to control sealer extrusion or lack of sealer material in the periapex, support the implementation of subtraction radiography in this study. Nevertheless, time differences observed between the present and previous clinical findings regarding the dimensional loss of a zinc oxide sealer are likely to result from factors that could not be replicated here, such as local acidic pH caused by inflammation (Urban et al., 2018) and/or foreign body reaction (Siqueira, 2005) in the periapical lesion.

Finally, throughout the observation timeline, the stability of the control gutta‐percha remained within the margins of experimental error (Figures 5, 6, 7). This stability confirms the dimensional changes registered for the endodontic sealers, and it is an important reference for this longitudinal evaluation. This is one of the strengths of the current study, as the use of a control is not often part of in vitro protocols (Rosa et al., 2010; Siboni et al., 2017).

Although this work employed the PRILE guidelines for laboratory studies (Nagendrababu et al., 2019), there is scope for improvement. Future research could for instance include systematically testing the type and pH of the immersion media to reflect the inflamed environment of a periapical lesion. This is a relevant aspect since calcium‐hydroxide‐based and calcium‐silicate‐based sealers modify the environmental pH by releasing hydroxyl ions. For instance, the pH and ion release from calcium‐hydroxide‐based sealers such as Apexit drop after 1 week most probably due to setting (Siqueira et al., 1995), whereas the alkalinized environment and extended ion release are shown for the calcium‐silicate‐based sealer BioRoot RCS for up to 6 months (Silva, Ferreira, et al., 2021; Urban et al., 2018). Additionally, such sealers are prone to higher dissolution in acidic environments, while resin‐based‐sealers are more resistant to low pH (Komabayashi et al., 2020). It would also be possible to use a printed bone‐like scaffold to support the teeth; this could be elaborated such that it included cellular and microbiological tests. Further improvement is also required to reduce the SE associated with the subtraction radiography method, and to a lesser extent with the analyses of 3D models.

## 5 CONCLUSIONS

The proposed in vitro model enabled following the dimensional status of endodontic sealers over 18 months on 3D models and, less effectively, through the use of subtraction radiography.

The largest dimensional changes were shown by Apexit Plus, followed by, to a much lesser extent, Tubliseal EWT and BioRoot RCS. AH Plus remained dimensionally stable throughout 18 months.

## AUTHOR CONTRIBUTIONS

Ankur Razdan, Ana R. Benetti, and Lars Bjørndal conceptualized the study and acquired the funding. Ankur Razdan, Ana R. Benetti, Azam Bakhshandeh, and Lars Bjørndal acquired the data. Tron A. Darvann developed the software and workflow for the volumetric analysis. Azam Bakhshandeh developed the workflow for the subtraction radiographic analysis. All authors analyzed and interpreted the data. Ankur Razdan, Ana R. Benetti, Lars Bjørndal, and Tron A. Darvann drafted the manuscript, which was critically revised by all authors.

## CONFLICT OF INTEREST

The authors declare no conflict of interest.

## Figures and Tables

**Figure A1:**
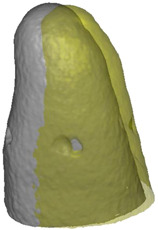
Example surfaces T0 (gray) and T1 (yellow) before registration

**Figure A2:**
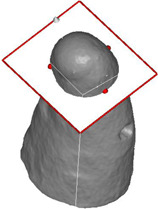
Three landmarks (red spheres) defining a plane for cutting a region of interest

**Figure A3:**
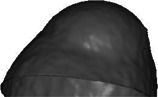
T0 after cutting

**Figure A4:**
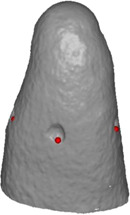
Three landmarks (red spheres) placed on “fiducials”

**Figure A5:**
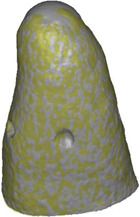
Surfaces after rigid landmark‐based registration followed by inverse closest point algorithm registration

**Figure A6:**
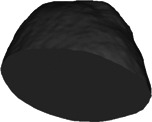
Surface after closing to get closed surface for volume calculation

**Figure A7:**
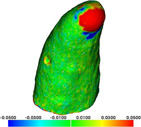
Color coded T0 surface according to distances (color bar in mm) indicating amount and spatial distribution of differences

## Data Availability

Data available on request to the authors.

## 1 DESCRIPTION OF “TOOTHREG” SOFTWARE

**Purpose**

The software carries out spatial registration of one or more surface scans of a tooth to a baseline scan of the same tooth. The software is tailored towards a situation where the temporal change in a small region of interest (ROI) is to be measured in terms of a volume change. We denote the baseline scan T0 and the following time instances as T1, T2, …. Ti …. Tm where m is the number of scans to be registered to the T0 scan. Figure A1 shows an example of two scans to be registered.

The implemented method is an ICP registration (iterated closest point algorithm) initialized by a rigid landmark transform using three manually roughly placed landmarks.

**Workflow**

(1) Three landmarks (l1, l2, l3) are placed manually on T0 to define a cutting plane to separate the ROI from the rest of the tooth surface (Figure A2). The ROI is the region where the change is to be measured (Figure A3).
(2) Three landmarks are also manually placed roughly on the “fiducials” (Figure A4) of each root surface T0 … Tm. These are used for the initial spatial registration of Ti to T0. This step was seen to be a necessary step before the application of ICP that otherwise could result in the ICP getting stuck in a local minimum.
(3) A landmark‐based rigid registration of T1 … Tm to T0 is carried out, using the landmarks defined in (2).
(4) An inverse ICP registration of T1 … Tm to the ROI of T0 is carried out. Figure A5 shows an example result.
(5) Cutting of T1 … Tm by the same plane as defined in (1) is carried out. Due to the registration in (4), the cutting is in exactly the same location on all surfaces.
(6) The ROI region (in all root instances) is closed by the plane (and any small holes in the surface are closed) (Figure A6) before the volumes (VT0 … VTi … VTm) are calculated.
(7) The volume change is calculated as VTi–VT0 where VT0 is the baseline volume.

All steps are implemented in landmarker (Darvann, 2008) (using VTK and Tcl/Tk), except the ROI closure in (6) which is accomplished using Autodesk Meshmixer.

In addition to these necessary steps, surfaces are inspected as an animation before registration and after registration using landmarker. Furthermore, registration error and amount of change are displayed as color‐coded surfaces, indicating the distance between surfaces in mm (Figure A7).
